# Clustering of Signs and Symptoms of Oral Diseases in a Colombian Population

**DOI:** 10.1016/j.identj.2022.06.007

**Published:** 2022-07-16

**Authors:** Ana Cristina Mafla, Falk Schwendicke

**Affiliations:** a School of Dentistry, Universidad Cooperativa de Colombia, Pasto, Colombia; b Escuela Internacional de Doctorado, Universidad Rey Juan Carlos, Madrid, Spain; c Department of Oral Diagnostics, Digital Health and Health Services Research, Charité Universitätsmedizin Berlin, Berlin, Germany

**Keywords:** Signs and symptoms, Dental caries, Periodontal disease, Bruxism, Cluster analysis, Multidimensional scaling analysis

## Abstract

**Objective:**

The aim of this study was to estimate disease pattern clusters and co-occurrences of oral signs and symptoms in a Colombian population.

**Methods:**

A cross-sectional study was carried out through a telephone survey amongst 1155 people registered in the telephone directory from Pasto, Colombia. The calls were made from July to November 2019. A 14-item self-report questionnaire about signs and symptoms related to oral diseases that included sociodemographic characteristics was employed. Descriptive and multivariable analyses such as hierarchical clustering, multidimensional scaling, and generalized linear models were used to determine co-occurrences in different sex and age strata.

**Results:**

Age- and condition-specific clusters of signs and symptoms were identified, while sex differences were limited. Calculus and denture sore mouth were related in 18- to 34-year-olds; tooth loss and calculus in 35- to 54-year-olds, and teeth holes or pits (dental caries) and dental abscess in those aged 55 years and older. We found stronger associations between periodontal disease (bleeding gums) and dental caries (odds ratio [OR], 2.484; 95% confidence interval [CI], 1.812–3.405; *P* < .001) as well as grinding/clenching and facial tension (OR, 7.162; 95% CI, 5.227–9.814; *P* < .001).

**Conclusions:**

Age-specific clustering of signs and symptoms and diagnostic patterns wer present in ths Colombian cohort.

## Introduction

A *sign* is an indicator of disease that the professional perceives; however, a *symptom* is a manifestation of disease apparent to the patients themselves. The sign is objective evidence of disease, while a symptom may be subjective.^1^ Signs and symptoms help the health professional to recognize and identify a current health problem and link it to a condition; moreover, some predict the state of health while others demonstrate the history of a patient. The ability to detect subtle signs and physical symptoms and differentiate them plays an equally important role in controlling a disease, as individuals use signs and symptoms to guide illness-regulation behaviors.

Every person has an individual perspective of illness^2^ and its signs and symptoms. Such a perspective may be influenced by complex factors such as culture or spirituality,^3^ socioeconomic conditions,^4^ or personality.^5^ However, the isolated assessment of specific signs and symptoms may not allow to comprehensively reveal the relation between them and their impact on health. Instead, a systematic assessment of the network co-occurring signs and symptoms, that is, their clustering,^6^ may be required. In dentistry, patients can self-assess certain signs and symptoms when they open their mouth or perceive other signs such as bad odor, overcrowding, or bleeding. Most prominently, they may experience pain of their teeth. Self-reported oral health (SROH) is widely used to determine oral health in surveys,^7^ and a wide range of studies confirmed SROH measures to be valid and reflecting the true clinical status.^8^, ^9^, ^10^, ^11^, ^12^ An important advantage of using self-reported oral health is the ease of collection (eg, via phone interviews instead of in-person assessments) and conduct (eg, collected by lay people rather than professionals).

The detection and evaluation of oral conditions reported as signs by clinicians or detected as symptoms by patients and understanding their complex relationship may allow to improve our capacity to interpretate them more effectively and act accordingly. The aim of this study was to define disease pattern clusters and co-occurrences of oral signs and symptoms in a Colombian population.

## Methods

### Study design, settings, and sample

A cross-sectional study was carried out through a telephone survey amongst people registered in the telephone directory from Pasto, Colombia. From this directory, systematic random sampling was performed, resulting in 4618 calls being made over 5 months (July to November 2019). Of these, 17% did not answer at all and 58% refused to participate because they did not have time or they did not want to give personal information. The survey response rate from calls made in this period was 25% (1155 individuals). In case the phone number belonged to a larger group of individuals, only one individual (usually the first person who answered the phone except when children or employees such as housemaids answered) was allowed to participate. Verbal consent was obtained before starting the survey.

Inclusion criteria were people aged 18 years or older, males or females, and having a valid telephone number. Calls with individuals who had a detectable cognitive impairment as well as calls with people that had interferences (bad telephone reception) in the moment of the survey were excluded.

### Measures and procedures

A questionnaire was defined with items about common signs and symptoms that were easily detectable; the instrument was then consented by dental clinicians from the dental clinic at Universidad Cooperativa de Colombia, Pasto, Colombia. This self-report 14-item questionnaire included oral conditions, such as “specks (aphthae),” “denture sore mouth,” “bleeding gums,” “calculus,” “teeth mobility,” “gingival recession,” “teeth holes and pits (dental caries),” “stabbing dental pain,” “dental abscess,” “tooth loss,” “dental trauma,” “overcrowding teeth,” “grinding/clenching,” and “facial tension”. For all items, the answer was coded as a dichotomous variable (“yes” and “no”). In order to obtain a higher number of participants, the telephone survey was designed to be as simple as possible. The completion time needed to complete the survey was between 4 and 6 minutes.

The questionnaire was pretested on 30 volunteers attending the dental clinic at Universidad Cooperativa de Colombia, Pasto, Colombia, to evaluate whether the questions were clear and could be answered quickly. To evaluate if the questionnaire was comprehensive, 3 “yes/no” questions and 1 “open” question were asked (“I understand the question,” “I understand but a change needs to made in this question,” “I do not understand this question,” “What change needs to be made in this question?”). After this pretesting process, words such as “aphthae”, “dental caries,” and “muscles contraction” were incorporated in the questionnaire.

Additional information on sociodemographic characteristics was obtained, including participants’ sex (coded as male and female according to World Health Organization definition)^13^; age (measured in years and classified into 3 groups (18–34 years, 35–54 years, and ≥55 years); socioeconomic status, categorized according to criteria based on housing quality indicators set by the Colombian government^14^ (coded as low, middle, and high); permanent residency (coded as Pasto [capital city of Nariño department] and other place); education (coded as none, primary, high school, and university); health insurance (coded as a dichotomous variable: “yes” and “no”); type of health insurance (coded as none, subsidized, and nonsubsidized); and religion (coded as a dichotomous variable: “yes” and “no”).

### Statistical analysis

Frequencies and percentages were estimated to determine the distribution of sociodemographic and sign and symptoms variables. An independent chi-square test was performed to examine differences among oral conditions in different demographic subgroups. Hierarchical age- and sex-stratified cluster analyses were employed to determine the relationship of sign and symptoms, with a virtual distance between 0 and 25 used for assessing the proximity of signs and symptoms. Multidimensional scaling was also age- and sex-stratified to find a representation of the least-squares among these variables in a 2-dimensional space (D1- and D2-axis) and to visualise relationships with lowest distortion and Kruskal's stress, implying similarity (suggested value of <0.10).^15^ Additionally, association of covariates with 3 outcomes (dental caries, periodontal disease assessed as bleeding gums, and bruxism) were estimated using generalized linear models. All signs and symptoms were entered into the models jointly with sex and age variables and then sequentially removed according to their association and *P* value (*P* < .2) through a backward elimination method. Missing data did not occur. *P* < .05 was considered statistically significant. SPSS v. 27 (IBM) was used for statistical analysis.

### Ethical approval

The protocol study and procedures were approved by Bioethics Sub-committee at Universidad Cooperativa de Colombia, Pasto, Colombia (Act No. SCBE05-18). They were in accordance with the 1964 Helsinki Declaration and its later amendments or comparable ethical standards. Informed consent was obtained from all participants included in the study.

## Results

### Sample characteristics

The sample comprised 1155 individuals from Pasto, Colombia. Amongst them, 464 participants were male (40.2%) and 691 were female (59.8%); 564 (48.9%) were 18 to 34 years old, 391 (33.8%) were 35 to 54 years old, and 200 (17.3%) were 55 years or older. Further, 597 (51.7%) and 558 (48.3%) belonged to a low and middle socioeconomic status, respectively; 1065 (92.2%) lived in Pasto (capital city of Nariño department), and 90 individuals (7.8%) resided in other places. Twenty-three participants (2.0%) did not have any education, 248 (21.5%) had studied at primary school, 506 (43.8%) finished high school, and 378 (32.7%) had a university degree. Overall, 1062 (91.9%) had a health insurance (44.6% subsidized and 47.3% nonsubsidized) and 1021 (88.4%) of the individuals reported having a religion.

### Oral signs and symptoms

There were differences in signs and symptoms between sexes. Males reported more dental trauma (26.5%) than females (12.3%; *P* < .001) and perceived more overcrowding (66.8%) than females (59.9%; *P* = .017). In general, the sign most often reported was “teeth holes or pits (dental caries)” (77.7%), and the least was “sore associated with dentures” (12.9%). There were statistical differences for almost all signs and symptoms amongst age groups, except for “specks” (aphthae), “grinding/clenching,” and “facial tension” (Table 1).

**Table 1:** Oral signs and symptoms.

Item	Question	Total N (%)	Males n (%)	Females n (%)	*P* value*	18–34 years n (%)	35–54 years n (%)	≥55 years n (%)	*P* value*
S1	**Do or did you have specks (aphthae) in your mouth?** ¿Usted tiene o ha tenido alguna vez fuegos (aftas) en la boca?	745 (64.5)	292 (62.9)	453 (65.6)	.361	351 (62.2)	254 (65.0	140 (70.0)	.139
S2	**Do or did you have soreness because of your dental dentures?** ¿Usted tiene o ha tenido ardor, dolor con sus prótesis totales?	149 (12.9)	55 (11.9)	94 (13.6)	.384	5 (0.9)	50 (12.8)	94 (47.0)	<.001
S3	**Do or did you have bleeding gums while brushing, flossing, or eating hard food?** ¿Usted tiene o ha tenido encías que sangran al cepillarse, usar la seda dental o comer comida dura?	788 (68.2)	321 (69.2)	467 (67.6)	.567	386 (68.4)	291 (74.4)	111 (55.5)	<.001
S4	**Do or did you have a crusty deposit around your teeth because of dental calculus or tartar?** ¿Usted tiene o ha tenido costras en sus dientes, debido a cálculo o sarro?	457 (39.6)	187 (40.3)	270 (39.1)	.676	155 (27.5)	187 (47.8)	115 (57.5)	<.001
S5	**Do or did you have tooth mobility when you bite?** ¿Usted tiene o ha tenido movilidad en sus dientes cuando muerde?	302 (26.1)	124 (26.7)	178 (25.8)	.715	86 (15.2)	124 (31.7)	92 (46.0)	<.001
S6	**Do or did you have recessions of your gums, ie, do your teeth look longer and/or can you see the roots of your teeth?** ¿Usted tiene o ha tenido encías que se retraen, ejemplo, usted mira que sus dientes se ven más largos, y/o puede mirar las raíces de sus dientes?	293 (25.4)	124 (26.7)	169 (24.5)	.385	93 (16.5)	117 (29.9)	83 (41.5)	<.001
S7	**Do or did you have visible holes or pits in your teeth?/Do or did you have dental caries?** ¿Usted tiene o ha tenido agujeros visibles en los dientes?/¿Usted tiene o ha tenido caries dental?	898 (77.7)	357 (76.9)	541 (78.3)	.588	418 (74.1)	319 (81.6)	161 (80.5)	.014
S8	**Do or did you have a stabbing pain in your teeth?** ¿Usted tiene o ha tenido dolor punzante en algún diente?	532 (46.1)	199 (42.9)	333 (48.2)	.076	214 (37.9)	199 (50.9)	119 (59.5)	<.001
S9	**Do or did you have a purulent abscess because you had a problem with a tooth?** ¿Usted tiene o ha tenido algún absceso con pus debido a que tuvo un problema con un diente dañado?	190 (16.5)	84 (18.1)	106 (15.3)	.214	69 (12.2)	80 (20.5)	41 (20.5)	.001
S10	**Have you lost a tooth before to other reasons than trauma/falls?** ¿Usted ha perdido dientes que no haya sido por golpes o caídas?	434 (37.6)	179 (38.6)	255 (36.9)	.565	130 (23.0)	159 (40.7)	145 (72.5)	<.001
S11	**Have or did you have cracked teeth because of trauma/falls?** ¿Usted tiene o ha tenido fracturas de dientes por golpes o caídas?	208 (18.0)	123 (26.5)	85 (12.3)	<.001	83 (14.7)	85 (21.7)	40 (20.0)	.015
S12	**Have you or did you have overcrowding teeth?** ¿Usted tiene o tuvo dientes torcidos?	724 (62.7)	310 (66.8)	414 (59.9)	.017	375 (66.5)	244 (62.4)	105 (52.5)	.002
S13	**Do you or did you grind or clench your teeth in sometime during the day or night?** ¿Usted aprieta o ha apretado o rechinado sus dientes en algún momento del día o de la noche?	322 (27.9)	137 (29.5)	185 (26.8)	.306	154 (27.3)	113 (28.9)	55 (27.5)	.857
S14	**Have or did you have pain because of facial tension (muscles contraction) when you wake up?** ¿Usted tiene o ha tenido dolor debido a tensión de la cara (contracción muscular) cuando despierta?	258 (22.3)	106 (22.8)	152 (22.0)	.735	120 (21.3)	84 (21.5)	54 (27.0)	.219

⁎ Derived from *χ*^2^. Differences between sex and age groups were tested.

### Clustering of signs and symptoms

As described, clustering of oral signs and symptoms for oral diseases was assessed by sex and stratified along the 3 different age groups (Figure). Bleeding gums (−0.231) and dental abscess (−0.214) were nearly related in males, and dental abscess (−0.271) and overcrowding teeth (−0.272) in females. Calculus (−0.009) and denture sore mouth (−0.006) were closely related in 18- to 34-year-olds; tooth loss (0.029) and calculus (0.169) in 35- to 54-year-olds, and teeth holes or pits (dental caries) (−0.068) and dental abscess (−0.057) in the group aged 55 years and older.

**Fig:**
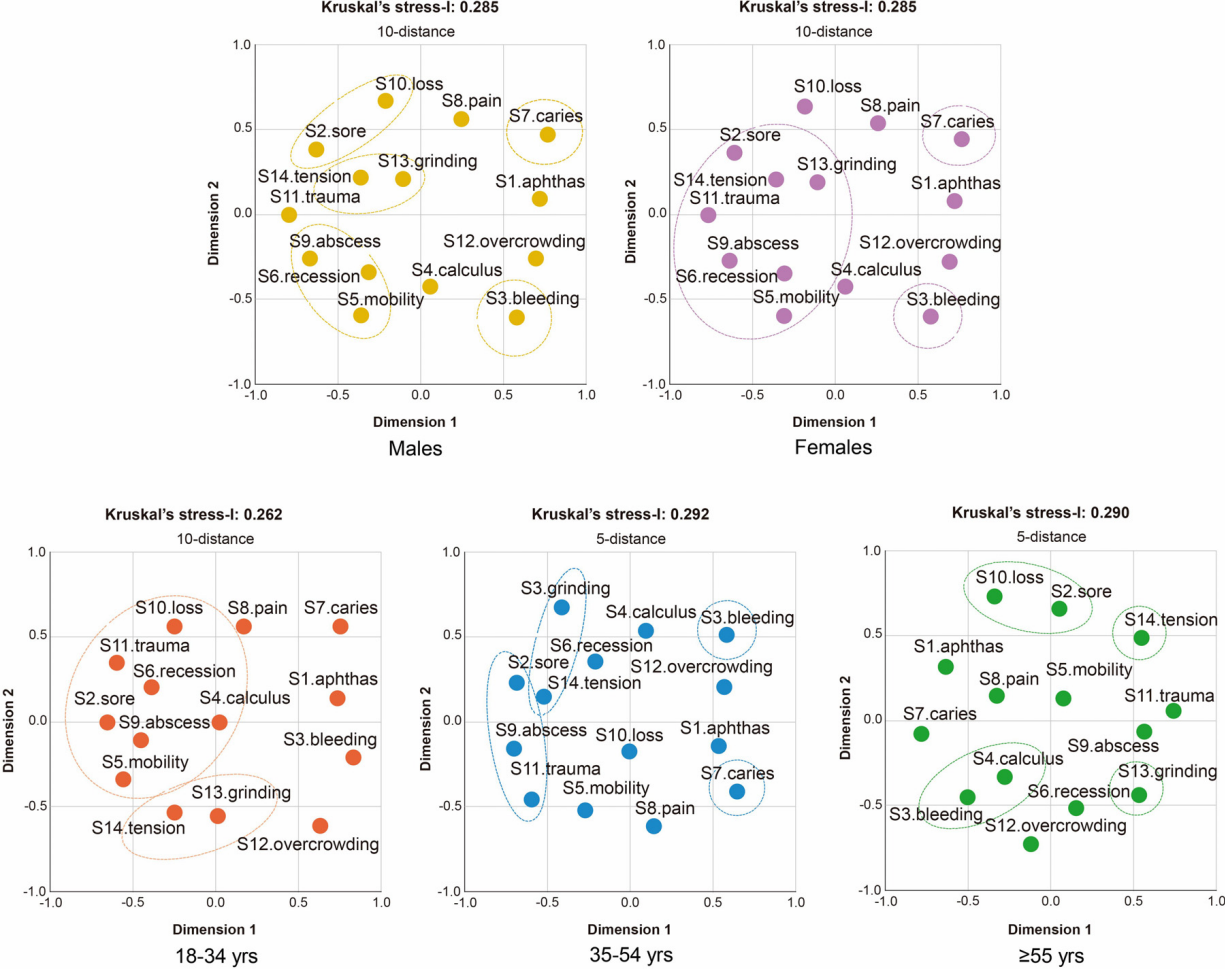
Multidimensional scaling analysis of signs and symptoms for oral disease co-occurrences by sex and age group.

S1, specks (aphthae); S2, denture sore mouth; S3, bleeding gums; S4, calculus; S5, teeth mobility; S6, gingival recession; S7, teeth holes or pits (dental caries); S8, stabbing dental pain; S9, dental abscess; S10, tooth loss; S11, dental trauma; S12, overcrowding teeth; S13, grinding/clenching; S14, facial tension.

### Association models

Tables 2 through 4 display the results of the association modelling. Teeth holes or pits (dental caries) had strong associations (*P* < .01) with bleeding gums (odds ratio [OR], 2.401; 95% confidence interval [CI], 1.759–3.277; *P* < .001), tooth loss (OR, 2.092; 95% CI, 1.380–3.170; *P* = .001), and overcrowding (OR, 1.973; 95% CI, 1.450–2.686; *P* < .001) (Table 2). Periodontal disease (bleeding gums), had strong associations with specks (aphthae) (OR, 1.986; 95% CI, 1.504–2.623; *P* < .001), teeth holes or pits (dental caries) (OR, 2.484; 95% CI, 1.812–3.405; *P* < .001), overcrowding (OR, 2.138; 95% CI, 1.620–2.822; *P* < .001), and age (OR, 0.961; 95% CI, 0.972−0.990; *P* < .001) (Table 3). Bruxism (grinding/clenching), had strong associations with stabbing pain (OR, 1.809; 95% CI, 1.342–2.438; *P* < .001), overcrowding (OR, 1.728; 95% CI, 1.261–2.369; *P* = .001), and facial tension (OR = 7.162; 95% CI, 5.227–9.814; *P* < .001) (Table 4).

**Table 2:** Association between presence of teeth holes or pits (dental caries) and different signs and symptoms.

Parameter	OR			
	Unadjusted [95% CI]	*P* value	Adjusted [95% CI]	*P* value
(Intercept)	-		0.417 [0.248–0.702]	.001
**Specks (aphthae)** (ref. no)	2.123 [1.601–2.815]	<.001	1.463 [1.075–1.991]	.016
**Denture sore mouth** (ref. no)	1.410 [0.901–2.207]	.133	0.448 [0.241–0.834]	.011
**Bleeding gums** (ref. no)	3.177 [2.384–4.232]	<.001	2.401 [1.759–3.277]	<.001
**Stabbing dental pain** (ref. no)	3.122 [2.292–4.252]	<.001	2.304 [1.658–3.202]	<.001
**Tooth loss** (ref. no)	2.283 [1.663–3.133]	<.001	2.092 [1.380–3.170]	.001
**Overcrowding teeth** (ref. no)	2.496 [1.882–3.311]	<.001	1.973 [1.450–2.686]	<.001
**Age** (*continuous*)	1.014 [1.005–1.024]	.004	1.014 [1.002–1.026]	.020

A general linear model was estimated with 13 oral signs and symptoms (specks [aphthae], denture sore mouth, bleeding gums, calculus, teeth mobility, gingival recession, stabbing dental pain, dental abscess, tooth loss, dental trauma, overcrowding teeth, grinding/clenching, and facial tension), sex, and age.

OR, odds ratio.

**Table 3:** Association between presence of periodontal disease (bleeding gums) and different signs and symptoms.

Parameter	OR			
	Unadjusted [95% CI]	*P* value	Adjusted [95% CI]	*P* value
(Intercept)			0.670 [0.429–1.048]	.079
**Specks (aphthae)** (ref. no)	2.587 [2.002–3.344]	<.001	1.986 [1.504–2.623]	<.001
**Calculus** (ref. no)	2.341 [1.785–3.070]	<.001	2.311 [1.698–3.144]	<.001
**Teeth holes or pits (dental caries)** (ref. no)	3.177 [2.384–4.232]	<.001	2.484 [1.812–3.405]	<.001
**Stabbing dental pain** (ref. no)	1.899 [1.471–2.451]	<.001	1.322 [0.988–1.768]	.060
**Dental trauma** (ref. no)	0.976 [0.708–1.346]	.881	0.724 [0.510–1.028]	.071
**Overcrowding teeth** (ref. no)	2.839 [2.197–3.668]	<.001	2.138 [1.620–2.822]	<.001
**Age** (*continuous*)	0.991 [0.983–0.999]	.024	0.961 [0.972–0.990]	<.001

A general linear model was estimated with 13 oral signs and symptoms (specks [aphthae], denture sore mouth, calculus, teeth mobility, gingival recession, teeth holes or pits [dental caries], stabbing dental pain, dental abscess, tooth loss, dental trauma, overcrowding teeth, grinding/clenching, and facial tension), sex, and age.

OR, odds ratio.

**Table 4:** Association between presence of bruxism (grinding/clenching) and different signs and symptoms.

Parameter	OR			
	Unadjusted [95% CI]	*P* value	Adjusted [95% CI]	*P* value
(Intercept)			0.100 [0.070–0.143]	<.001
**Specks (aphthae)** (ref. no)	2.028 [1.519–2.709]	<.001	1.501 [1.087–2.071]	.013
**Stabbing dental pain** (ref. no)	2.066 [1.591–2.684]	<.001	1.809 [1.342–2.438]	<.001
**Tooth loss** (ref. no)	1.057 [0.811–1.377]	.684	0.700 [0.513–0.954]	.024
**Overcrowding teeth** (ref. no)	1.981 [1.492–2.630]	<.001	1.728 [1.261–2.369]	.001
**Facial tension** (ref. no)	7.588 [5.597–10.285]	<.001	7.162 [5.227–9.814]	<.001

A general linear model was estimated with 13 oral signs and symptoms (specks [aphthae], denture sore mouth, bleeding gums, calculus, teeth mobility, gingival recession, teeth holes or pits [dental caries], stabbing dental pain, dental abscess, tooth loss, dental trauma, overcrowding teeth, and facial tension), sex, and age.

OR, odds ratio.

## Discussion

Based on self-reported signs and symptoms, we found that 77% of the sampled population perceived to have dental caries, 68% bleeding gums and 40% calculus (ie, signs of periodontal disease), 46% stabbing pain and 16% dental abscess (ie, signs and symptoms of pulpal and periapical diseases), and 28% tooth grinding (ie, bruxism). In other populations where the prevalance of oral diseases were evaluated a number of conditions similarly prevailed. For instance, the prevalence rates of dental caries were between 47% and 84% in Norway, Kuwait, and Brazil.^16^, ^17^, ^18^ The presence of periodontal disease varied from 43% to 91% in Nepal residents.^19^ Additionally, 27% of the Taiwanese had pulp and periapical diseases,^20^ while 14% and 37% of the Brazilians and Turkish, respectively, in this range of age, exhibited bruxism.^21^^,^^22^

Co-occurrence of oral conditions are likely and have been found by the present study, too, possibly routed in common risk factors triggering not one but several conditions. When patients attend a routine dental visit, they usually report signs and symptoms they have perceived for a while; assessing these signs and symptoms and their co-occurrence may help clinicians to more comprehensively evaluate and understand patients’ oral health status. Our descriptive analysis showed few variations in clustering by sex and indicated marked differences in clustering and association by age as well as condition (dental caries, periodontal disease assessed as bleeding gums, and bruxism). Age- and condition-specific assessments should be considered in clinical routine.

A number of findings require discussion. We observed that facial tension and grinding consistently clustered in all ages and independently of other conditions as symptoms of bruxism. Showing consistent associations across subgroups highlights the robustness of our findings and indicates that clustering analyses can yield worthwhile results that are in agreement with clinical reasoning. However, interestingly, in females facial tension and grinding clustered with other signs and symptoms, while in male those were independent from others. These symptoms of bruxism are related to stress and anxiety,^23^ which may be more frequently reported in women.^24^^,^^25^ They may influence not only this condition but other diseases because of their biopsychosocial components.^26^

For other conditions, age-specific clustering was apparent. For example, in early adulthood (18–34 years), dental conditions are unlikely to have occurred in a majority of individuals; the identified cluster involving tooth loss, dental abscess, tooth mobility, and denture soreness is likely a marker for high-risk individuals experiencing a wide range of conditions at young age. Identifying these individuals via assessing the co-occurrence of signs and symptoms would be worthwhile to address the needs of these particular individuals. In this respect, the literature reports an association between dental caries and oral *Candida*,^27^ which may be related to signs and symptoms of dental abscess and denture soreness.

In adults (35–54 years), we found an association between signs of dental caries (dental holes or pits) and bleeding gums, likely as periodontitis onset happens in this age group for most individuals, while both diseases share common risk factors like biofilm formation and a sugary diet^28^ and they may emerge in response to nutritional imbalances in the microbiota.^29^ In general, our analyses highlight the association among dental plaque (measured via calculus), dental caries,^30^^,^^31^ and periodontal disease.^32^^,^^33^ Moreover, a possibly impaired immune system may accelerate dental plaque accumulation ^34^ in econiches. In older adults (≥55 years), clustering analysis confirmed the expected association of dental conditions (mainly periodontal disease), tooth loss, and denture soreness.^35^

Based on our models’ findings, dental caries and oral aphthae were associated, possibly via bacterial infections.^36^ Generally, microbiota may explain the link between different signs and symptoms. Besides, tooth loss may be, as outlined, a marker of poor oral health, that is, chronic inflammation and infection of the pulp^37^ as a result of dental caries, and may occur alongside missing teeth, lost due to similar processes earlier. Dental caries and biofilm accumulation have also been reported to be more prevalent in individuals with overcrowding.^38^ The association between calculus and periodontal disease has been well established,^39^ and a higher level of stress has been associated not only with sleep bruxism^40^ but with aphthae.^41^

Overall, assessing sets of signs and symptoms seems useful to more comprehensively map individuals’ oral health burden and treatment needs and to distinguish high- from low-risk individuals. Future studies should link the underlying risk factors to these networks of signs and symptoms to deepen our understanding of the genesis of the observed co-occurrences and how to address the disease clusters. Also, replication of our analyses in other populations should be attempted. Epidemiologic surveys may want to consider to complement clinical assessments with comprehensive signs and symptoms mapping, as this may allow to systematically assess the interdependence of diseases and their subjective impact on individuals.

This study comes with a number of limitations. First, the collection of data used a self-reporting scheme. While self-reporting may be valid for collecting signs and symptoms, it may not fully allow to replicate clinical assessment of oral conditions. Future studies should add a clinical examination element for triangulation purposes. Additionally, self-reports by phone may be may prone to different bias^42^ than those by in-person interviews, something we accepted given the ease of data collection and random population-representative sampling. Second, we did not employ a validated collection instrument, as our aim was to explore the option of gauging signs and symptoms, and as a validated instrument does not exist, our study may help to build a theoretical foundation to develop such instrument in the future. Third, our cross-sectional design and the resulting analyses do not allow to infer causal relationships^43^; using cohort data may permit to unravel the timing of the occurrence of signs and symptoms and thereby yield deeper insights towards causality. Last, our sample population was representative for Pasto, Colombia; signs and symptoms and their associations may differ in other populations and we cannot claim generalizability.

## Conclusions

Dentists should assess co-occurring signs and symptoms of diseases, comprehensively covering their complex associations. Age-specific clusters may warrant age-specific diagnostic patterns.

## Conflict of interest

None disclosed.
